# Balancing macronutrient intake in a mammalian carnivore: disentangling the influences of flavour and nutrition

**DOI:** 10.1098/rsos.160081

**Published:** 2016-06-15

**Authors:** Adrian K. Hewson-Hughes, Alison Colyer, Stephen J. Simpson, David Raubenheimer

**Affiliations:** 1 WALTHAM Centre for Pet Nutrition, Melton Mowbray, Leicestershire LE14 4RT, UK; 2 The Charles Perkins Centre and School of Biological Sciences, University of Sydney, Sydney, New South Wales 2006, Australia; 3 Faculty of Veterinary Science, University of Sydney, Sydney, New South Wales 2006, Australia

**Keywords:** carnivore, geometric framework, food selection, hedonic, macronutrient, cats

## Abstract

There is a large body of research demonstrating that macronutrient balancing is a primary driver of foraging in herbivores and omnivores, and more recently, it has been shown to occur in carnivores. However, the extent to which macronutrient selection in carnivores may be influenced by organoleptic properties (e.g. flavour/aroma) remains unknown. Here, we explore the roles of nutritional and hedonic factors in food choice and macronutrient balancing in a mammalian carnivore, the domestic cat. Using the geometric framework, we determined the amounts and ratio of protein and fat intake in cats allowed to select from combinations of three foods that varied in protein : fat (P : F) composition (approx. 10 : 90, 40 : 60 and 70 : 30 on a per cent energy basis) to which flavours of different ‘attractiveness’ (fish, rabbit and orange) were added. In two studies, in which animal and plant protein sources were used, respectively, the ratio and amounts of protein and fat intake were very consistent across all groups regardless of flavour combination, indicating regulation of both protein and fat intake. Our results suggest that macronutrient balancing rather than hedonistic rewards based on organoleptic properties of food is a primary driver of longer-term food selection and intake in domestic cats.

## 1. Introduction

The mammalian order Carnivora is of particular interest for the study of the relationship between food selection and nutrition for at least two reasons. First, it lends itself to the study of the evolution of nutritional strategies because, although ancestrally carnivorous, today the order includes species with a diversity of dietary habits. A second reason why it is important to study nutrition and food intake in Carnivora is that it includes our major companion animals, cats and dogs. To date, five species of Carnivora have been the subject of studies aimed at understanding their nutritional regulatory responses. Two of these, the domestic cat, *Felis silvestris catus* [1] and the mink, *Neovison vison* [2,3] consume diets composed almost exclusively of animal tissue (so-called ‘hypercarnivores’; [4]), whereas the grizzly bear, *Ursus arctos horribilis* [5–8] and the domestic dog, *Canis lupus familiaris* [9–11] are more omnivorous in their dietary habits. Yet another species, the endangered Giant Panda, *Ailuropoda melanoleuca*, which today is principally herbivorous, structures its annual pattern of migration around macronutrient and calcium availability [12].

The results of these studies add to the growing body of evidence showing that animals across many taxa including insects, birds, fish and mammals are able to regulate and balance their intake of multiple nutrients, particularly the macronutrients protein, fat and carbohydrate, by adjusting their intake of the foods available to them (either naturally available or provided experimentally; [13–21]).

Historically, research on factors affecting food selection and food intake in domestic cats has largely focused on organoleptic properties of foods (e.g. flavour, aroma, texture and temperature; [22–25]), particularly as studies investigating food choice based on nutritional properties seemed somewhat inconclusive. For example, Cook *et al.* [26] concluded that ‘kittens did not regulate in a consistent manner their intake of protein’ on the basis that they did not select for (i.e. eat significantly more of) a high-protein food compared with a lower protein food when offered a choice. Similarly, kittens given a choice of foods containing 4 or 6 g kg^−1^ threonine (where 6 g kg^−1^ threonine was reported to be the minimal requirement for kittens determined in a separate study [27]) ate similar amounts of both foods—leading the authors to conclude that ‘the cats failed to choose a diet that contained an adequate threonine concentration’ [28]. That cats did not appear to select foods based on the nutrient content was in keeping with the view that carnivores would have no need to regulate their nutrient intake as their natural food (i.e. prey) would be relatively consistent in nutrient composition and hence provide the right balance of nutrients to the predator [29].

It is now becoming clear that not only do prey species vary considerably in protein and fat content [4,30–32], but also predators do alter their food selection and intake in relation to the nutrient composition of foods. Thus, studies on mammalian carnivores (i.e. mink and domestic cats; [1,2]) and invertebrate predators [18,31] have provided evidence of macronutrient intake regulation. The diet of feral cats has been shown to comprise the same high proportion of energy from protein and fat [33] as that composed by domestic cats in a research setting [1] that have never consumed prey, suggesting that regulation of macronutrient intake is sufficiently fundamental to transcend the organoleptic differences between hunted prey and commercial cat foods. Here, we attempt to disentangle the role of nutritional and organoleptic properties of food on food selection and intake in domestic cats using the geometric framework (GF) for nutrition [15,21]. We tested the hypothesis that the amounts of protein and fat ingested by cats would not be different when they were allowed to select from combinations of three foods that varied not only in protein : fat (P : F) composition but also with different added flavours/aromas.

## 2. Material and methods

### 2.1. Animals and housing

Adult domestic short hair cats (*F. silvestris catus*) of both sexes, bred and housed at the WALTHAM Centre for Pet Nutrition (WCPN), Melton Mowbray, Leicestershire, UK, participated in these diet selection experiments. Unless otherwise stated, cats were housed and fed individually throughout each experiment in purpose-built, behaviourally enriched lodges (w × d × h: 1.1 m × 2.5 m × 2.1 m), were socialized as a group for approximately 2 h each day (08.30–10.30 h) and had access to drinking water at all times. The cats were housed in social groups when not participating in experiments.

### 2.2. Experimental protocols

In this series of experiments, the first aim was to determine whether the addition of exogenous flavours/aromas (fish, rabbit and orange) altered the intrinsic palatability (‘attractiveness’) of the experimental foods from the cat's perspective in a 20 min, no-choice feeding test (experiment 1). We then tested the ability of cats to maintain a consistent intake of protein and fat when offered a choice of foods with different protein and fat levels in which the inherent flavours/aromas and the macronutrient content of the foods were ‘disguised’ using the fish (positive), rabbit (neutral) and orange (negative) flavours (experiments 2 and 3).

#### 2.2.1. Experiment 1

Three flavours were selected (rabbit (Quest International, 1411 GP Naarden, The Netherlands), fish (Firmenich UK Ltd., Middlesex, UK) and orange (Firmenich UK Ltd., Middlesex, UK)) based on the expectation that cats would be able to discriminate between them, i.e. they would result in differences in intake when added to a food.

Recipes were formulated for three foods to provide predicted per cent energy from protein and fat (PE : FE) of 10 : 90, 40 : 60 and 70 : 30. As the aim of the subsequent choice studies (experiments 2 and 3) was to determine whether the macronutrient content of the food influenced food choice and macronutrient intake, the foods were designed to represent extremes of protein content with the 10 : 90 food being below the National Research Council (NRC) recommended allowance (RA; 50 g 1000 kcal^−1^) and the 40 : 60 and 70 : 30 foods being approximately two and three times the RA, respectively [34]. The foods were prepared fresh each day by mixing appropriate amounts of skinless chicken breast (steam sterilized in cans at Mars Petcare, Melton Mowbray, UK), lard (melted in a microwave) with vitamins and minerals added to meet nutritional recommendations for adult cats ([34]; table 1). Textural differences between the foods were reduced by homogenizing the ingredients in an electric food mixer and including varying amounts of carob solution (1.5%) to achieve a similar texture/consistency (similar to porridge). For each food PE : FE level (i.e. 10 : 90, 40 : 60 and 70 : 30), four flavours were produced—rabbit (used at 0.06% w/w), fish (used at 1.5% w/w), orange (used at 0.03% w/w of a 19% w/w mixture of orange oil in sunflower oil) and ‘unflavoured’ (i.e. no exogenous flavour added). The flavours were added at concentrations that were just detectable by humans by aroma and taste and were presumed to be detectable by cats.

**Table 1.** Ingredients and analysed nutrient contents of the foods used.

	experiments 1 and 2			experiment 3		
	10 : 90^a^	40 : 60	70 : 30	10 : 90	40 : 60	70 : 30
ingredients (g kg^−1^ as fed)						
skinless chicken breast	75.7	304.7	533.7	35.3	35.2	35.5
soy protein isolate	—	—	—	8.8	70.3	131.6
lard (pork fat)	89.0	55.5	22.0	69.2	43.0	16.8
carob solution (1.5%)	814.3	628.8	438.3	844.5	819.9	795.1
vitamin mix^b^	11	6	3	20	15	10
mineral mix^c^	10	5	3	20	15	10
taurine	—	—	—	0.7	0.6	0.5
L-methionine	—	—	—	1.5	1.0	0.5
analytical values (% as fed)						
moisture	86.5	84.0	80.5	88.9	85.5	83.2
protein	1.8 [17]	8.4 [90]	15.1 [163]	1.6 [32]	6.3 [84]	12 [170]
fat	10.9 [103]	6.4 [69]	3.5 [38]	4.9 [97]	5.5 [74]	2.3 [33]
carbohydrate	0	0.5 [5]	0.2 [2]	0	0	0.5 [7]
PME (kJ 100 g^−1^)	440.6	389.9	387.9	234.7	312.5	307.5
PE%	7	36	65	13	34	68
FE%	93	62	34	87	66	29
CE%	—	2	1	—	—	3

^a^Approximate % distribution of protein energy (PE) : fat energy (FE) in each food. These rounded values have been used as descriptors of the diets within this paper. The carbohydrate content was expected to be negligible as none was added, but since it is calculated by difference (100 – %moisture + %protein + %fat + %ash + %crude fibre), values for carbohydrate energy (CE) were derived for some foods. Because the foods were not commercially prepared products, Atwater factors of 16.7, 37.6 and 16.7 kJ g^−1^ were used for protein, fat and carbohydrate, respectively, to calculate the predicted metabolizable energy (PME) [34]. The values in square brackets [ ] for protein, fat and carbohydrate are g 1000 kcal^−1^.

^b^Composition (g kg^−1^ mix): retinol acetate (vitamin A), 0.99; vitamin D_3_, 0.9 mg kg^−1^; α-tocopheryl acetate (vitamin E), 17.8; vitamin B_2_ (riboflavin), 0.5; d-calcium pantothenate, 0.04; vitamin B_6_ (pyridoxine-HCl), 0.4; folic acid, 0.1; vitamin B_12_, 3.0 mg kg^−1^; biotin, 0.01; taurine, 443.4; methionine, 535.9.

^c^Composition (g kg^−1^ mix): CaCO_3_, 292.6; CaHPO_4_, 438.4; NaCl, 19.5; K_2_HPO_4_, 188.9; MgSO_4_, 47.1; ferric citrate, 10.0; CuSO_4_, 0.39; MnSO_4_, 0.72; ZnCl_2_, 2.3; sodium selenite 0.004.

Twenty-four adult cats (12 male and 12 female) that had no prior experience of the experimental foods or flavours were placed in individual feeding stations (0.5 m × 0.5 m × 0.5 m) for 20 min (09.00–09.20 h) and offered 150 g of one of the PE : FE–flavour foods per day. Each cat was exposed to each of the 12 PE : FE–flavour combination twice in a randomized sequence over 24 days. Apart from this 20 min period, cats were housed together in social rooms and were provided with standard commercially available wet food (equivalent to 300 g per cat) overnight (from 15.30 to 08.30 h the following morning).

#### 2.2.2. Experiment 2

Twenty-seven cats (16 neutered males (MN), 9 neutered females (FN) and 7 entire females (FEn), 4.4 ± 2.9 years; 4.18 ± 0.79 kg) that were naive to the foods and flavours were split into three groups (*n* = 9 per group) balanced as much as possible for gender, neuter status and age. The foods were made as in experiment 1 to give predicted PE : FEs of 10 : 90, 40 : 60 and 70 : 30 with fish, rabbit and orange flavoured versions produced for each food (i.e. nine different PE : FE–flavour combinations were produced). Each group of cats was allocated to receive a particular three-food combination (table 2). Cats that lost more than 15% of their initial body weight due to poor food intakes were removed from the trial and data were excluded from the analyses. This included two cats from group 1 and two from group 3. The feeding protocol consisted of two phases—a monadic ‘learning’ phase and an experienced self-selection (ESS) phase.

**Table 2.** % Protein energy : fat energy content (PE : FE) and flavour combinations of the foods offered to each group of cats in experiments 2 and 3.

group	PE : FE–flavour combinations		
1	10 : 90 + fish	40 : 60 + rabbit	70 : 30 + orange
2	10 : 90 + orange	40 : 60 + fish	70 : 30 + rabbit
3	10 : 90 + rabbit	40 : 60 + orange	70 : 30 + fish

##### 2.2.2.1. Phase 1: monadic learning.

During this phase, each cat received a single product each day, with the three experimental foods appropriate for the group fed in daily rotation for a total of 30 days (i.e. 10 exposures to each of the three foods). Each cat received 200 g of food in the morning (10.30 h), which was replaced with a fresh 300 g of the same food in the afternoon (15.00 h) and left in the lodge with the cat overnight (removed at 08.30 h the following morning). Any uneaten food was weighed as food was replaced at 15.00 and 08.30 h and food intake was calculated as the difference between the mass of food offered and the mass of food remaining. Typical evaporative losses from all three diets was 6–7% of the initial weight of the food over a period equivalent to the longest time over which the diets were presented to the cats (i.e. overnight). As there were no differences in evaporative losses between the three diets, all food intakes are based on data without any correction for evaporative loss. From the food intake data and the proximate analysis values for the foods, the amounts of protein and fat consumed by the cats were calculated.

##### 2.2.2.2. Phase 2: experienced self-selection.

For the final 7 days, each cat was given simultaneous access to the three foods according to their group in three separate bowls. Food was replaced twice per day as described for phase 1; 200 g of each food was offered in the morning (i.e. 600 g of food in total), which was replaced with a fresh 200 g of each food in the afternoon (i.e. 600 g of food in total). The position of the foods was rotated each day to avoid positional bias. Food intakes were recorded every time food was replaced as described in phase 1 to allow the amount of protein and fat consumed to be calculated.

#### 2.2.3. Experiment 3

This experiment was performed to investigate whether using a non-animal protein source (soya protein isolate) would influence the macronutrient intake of the cats. The experimental foods were very similar to experiment 2 but with the recipes modified by the inclusion of increasing amounts of powdered soya protein isolate (ICN, Irvine, CA, USA) and a base inclusion of chicken breast (table 1). The 27 cats (16 MN and 11 FN, 2.4 ± 0.5 yr; 4.30 ± 1.36 kg) that took part in this experiment were naive to the foods and flavours and were split into three groups as in experiment 2 (table 2). Cats that lost more than 15% of their initial body weight due to poor food intakes were removed from the trial and data were excluded from the analyses. This included one cat from group 1 and three from group 2.

A second objective was to determine whether the ‘learning’ phase described in experiment 2 was necessary for cats to be able to regulate their macronutrient intake. This was tested by introducing a 7-day ‘naive’ self-selection phase (NSS, phase 1) before the ‘monadic learning phase’ (phase 2) which could be compared with the ESS phase (phase 3). The details for phases 1 and 3 were as described for the ESS phase in experiment 2. The details for phase 2 were as described for monadic learning in experiment 2 except that after eight cycles (24 days) cats were transferred to standard food for 12 days until supplies of soy isolate powder were received. Following this break, cats were given five cycles (15 days) of exposure to each food resulting in a total of 13 exposures to each of the three foods.

### 2.3. Data analysis

#### 2.3.1. Experiment 1

The main objective of this experiment was to determine whether the amount of food consumed varied by the flavour (i.e. fish, rabbit, orange or no added flavour) when cats were provided with a single food containing one of three levels of P : F. The intake (grams) was analysed by multi-factor ANOVA with cat, P : F content, flavour and their interaction as factors. Statistical analysis was performed in StatGraphics Plus v. 4.1 (Manugistics Inc., Maryland, USA).

#### 2.3.2. Experiments 2 and 3

The main objective of these experiments was to determine whether the total daily intake of protein and fat was different when provided with a choice of foods containing different P : F levels and different flavours/aromas. For the self-selection phases of each experiment, total daily intake of protein (grams) and fat as well as total energy intake (kilojoules) were the primary measures analysed and were log_10_ transformed prior to analysis. Mixed effects models were fitted for each measure with cat as the random effect and group (i.e. food combination offered) as a fixed effect. Differences in each measure between groups were compared using Tukey HSD tests.

To compare differences in total daily protein and fat intake between self-selection phases within and between experiments 2 and 3, mixed effects models were fitted with phase nested in cat as the random effect. Phase, group and their interaction were fitted as fixed effects. Phase means were compared using an overall family-wise error rate of 5%.

The total daily intake of each food in the self-selection phases was analysed as a secondary measure. A linear mixed effects model was fitted for the daily intake (in grams) of each food with cat as the random effect and PE, flavour and their interactions as fixed effects.

For all measures, means and differences between means are reported or plotted with family-wise adjusted 95% confidence intervals (CIs), after back transformation from log_10_ values where appropriate. Statistical analyses were performed using v. 2.15 of the statistical software package R (http://www.r-project.org/), using the *nlme* and *multcomp* libraries.

## 3. Results

### 3.1. Experiment 1

**Figure 1.**
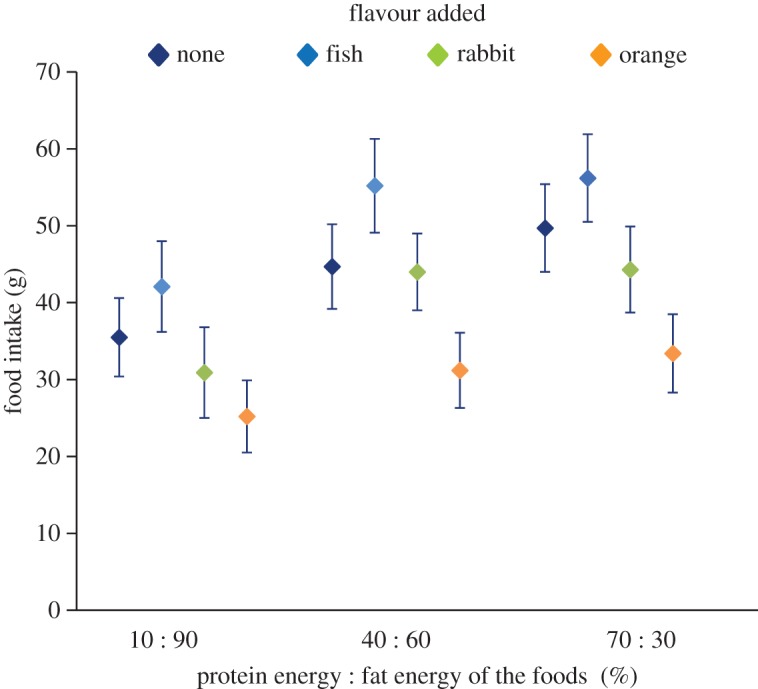
Mean intake (±s.e.m.) of each food (with fish, rabbit, orange or no flavour (none) added) offered individually to 24 cats in 2 × 20 min intake tests in a randomized order over 24 days (experiment 1).

Cats were able to distinguish between the flavours added to the foods as indicated by a significant difference between the mean intakes of the 12 nutrition–flavour combinations (*p *< 0.001). Figure 1 shows the mean intake over two exposures to each product and indicates the relative ranking of the flavours was fish > rabbit = none > orange across all PE : FE levels, which we interpret as a positive ingestive effect of the fish flavour, a ‘neutral’ effect of the rabbit flavour and a negative/aversive effect of the orange flavour.

### 3.2. Experiment 2

**Figure 2.**
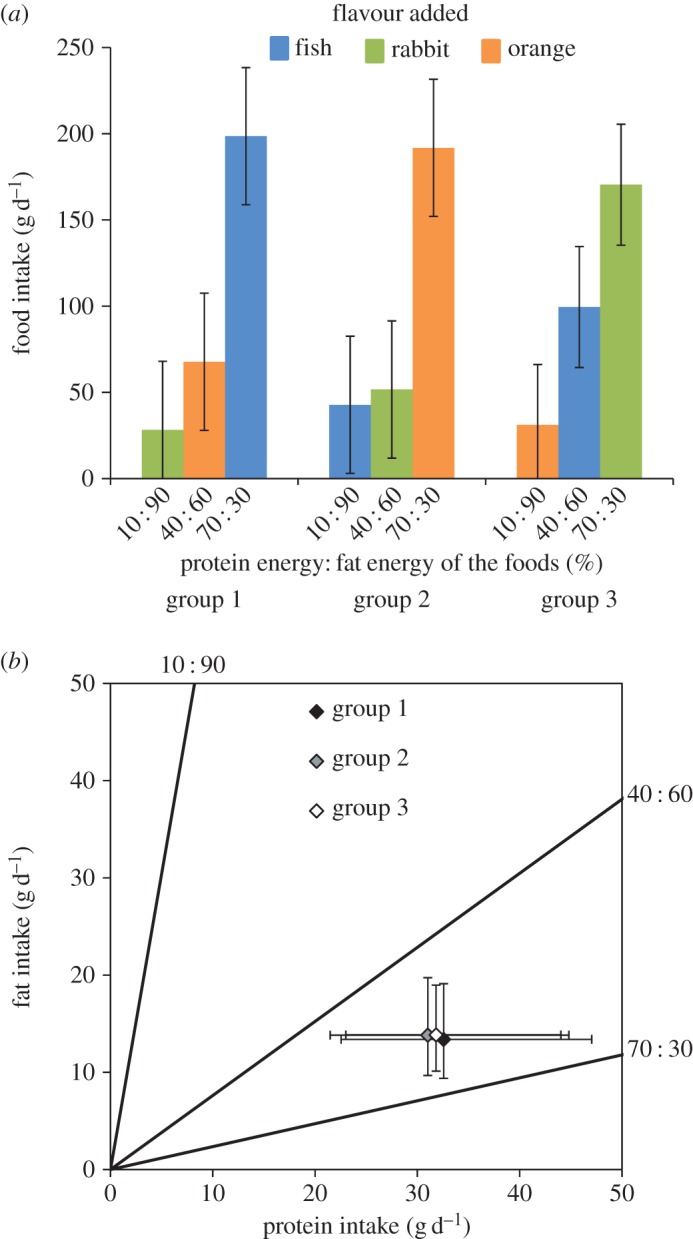
(*a*) Mean daily intake (with 95% CIs) of each the three foods offered simultaneously to three groups of cats in the self-selection phase of experiment 2. (*b*) Geometric representation of protein and fat intake regulation by cats in all three groups. Mean daily protein and fat intake (with 95% CI) is plotted for each group of cats as a result of the amounts of each food consumed above. The black lines (nutrient rails) represent the P : F balance of each of the foods provided.

When offered a choice of three foods, the pattern of food intake was similar across all three groups with intake of the 70 : 30 food being significantly greater than the 10 : 90 and 40 : 60 foods within each group irrespective of added flavour (figure 2*a*). Thus, for group 1, there was an average difference in intake of 130.8 g (95% CI 95.4, 166.3 g; *p *< 0.0001) between the 70 : 30 food and the 40 : 60 food (i.e. the food with the next highest intake in that group). For cats in groups 2 and 3, the differences in intake between the 70 : 30 and 40 : 60 foods were 140.2 g (95% CI 104.7, 175.6 g; *p *< 0.0001) and 71.0 g (95% CI 39.7, 102.2 g; *p *< 0.0001), respectively.

When the intakes of protein and fat were plotted, it was very clear that the mean intakes of these macronutrients converged to very consistent amounts across all three groups (figure 2*b*). Cats in group 1 ate 32.6 g protein d^−1^ (95% CI 22.5, 47.0 g) and 13.4 g fat d^−1^ (95% CI 9.4, 19.1 g), cats in group 2 consumed 31.0 g protein d^−1^ (95% CI 21.5, 44.8 g) and 13.8 g fat d^−1^ (95% CI 9.7, 19.7 g) and cats in group 3 ate 31.8 g protein d^−1^ (95% CI 23.0, 44.0 g) and 13.9 g fat d^−1^ (95% CI 10.1, 19.0 g). The resulting amounts of protein and fat ingested per day were not significantly different between the three groups of cats. The largest difference in protein intake was 1.5 g (95% CI −11.5, 23.2 g; *p *= 0.973) between groups 1 and 2 and the largest difference in fat intake was 0.5 g (95% CI −5.5, 7.5 g; *p *= 0.984) between groups 1 and 3.

### 3.3. Experiment 3

Overall food intake was lower in the NSS phase than the ESS phase which is reflected in a significant effect of phase (*p *< 0.0001), but not choice of foods offered (*p *= 0.400) on total energy intake. Cats consumed approximately half as much energy during the NSS phase compared with the ESS phase with a mean difference of 509.8 kJ (95% CI 406.2, 613.4 kJ; *p *< 0.0001).

**Figure 3.**
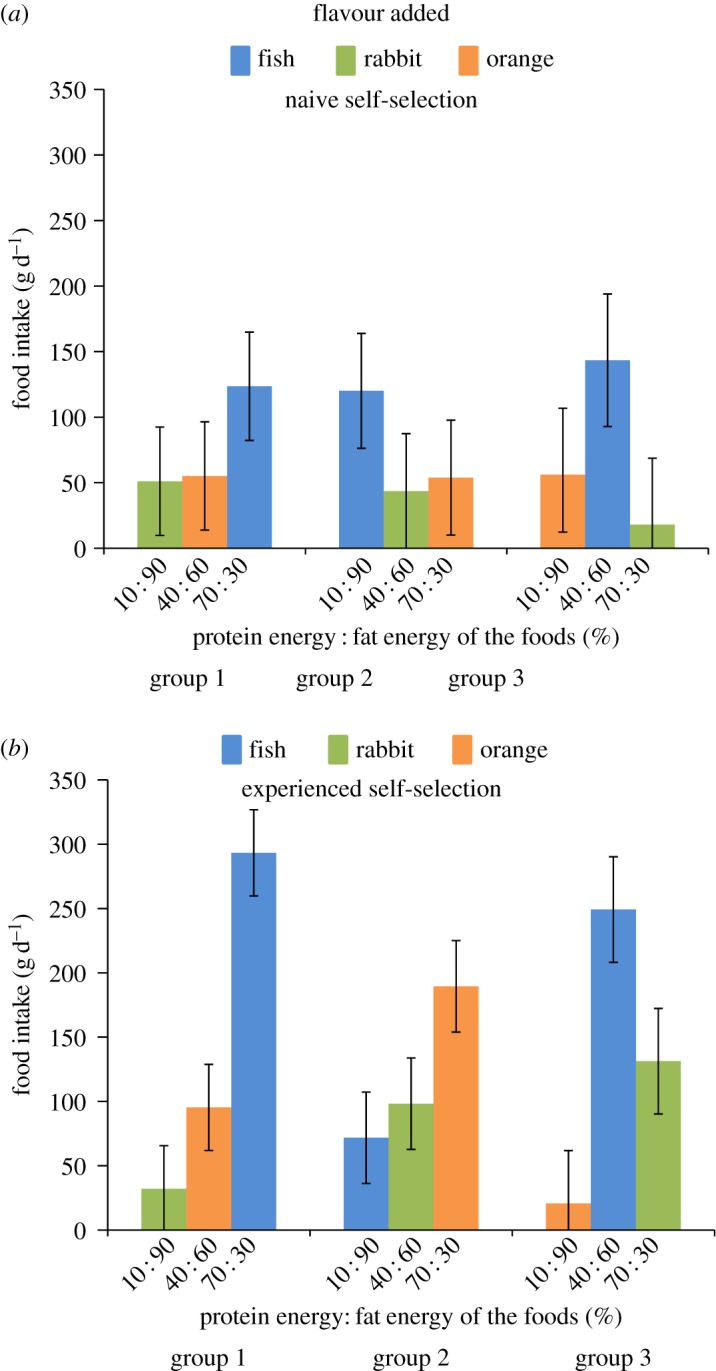
Mean daily intake (with 95% CI) of each of the three foods offered simultaneously to three groups of cats in the naive (*a*) and experienced (*b*) self-selection phases of experiment 3.

During the NSS phase, flavour appeared to dominate the food choice of the cats with the fish-flavoured foods having a significantly greater intake within each group regardless of the P : F content of the food (figure 3*a*). The intake of the fish-flavoured food was on average 68.5 g (95% CI 32.5, 104.5 g; *p *< 0.0001), 66.2 g (95% CI 28.0, 104.4 g; *p *< 0.0001) and 87.2 g (95% CI 43.2, 131.3 g; *p *< 0.0001) greater than the food with the next highest intake for groups 1, 2 and 3, respectively (figure 3*a*). Following the learning phase, the intake of the fish-flavoured food remained significantly greater than the other foods for cats in both group 1 (i.e. 197.9 g (95% CI 156.6, 239.2 g) greater intake compared with the food with next highest intake, 40 : 60 + orange; *p *< 0.0001) and group 3 (117.9 g (95% CI 67.3, 168.5 g) greater intake compared with 70 : 30 + rabbit; *p *< 0.0001) (figure 3*b*). By contrast, the cats in group 2 markedly shifted their pattern of food intake such that in the ESS phase the intake of the orange-flavoured food (with the highest protein content, 70 : 30) was now significantly greater than the other two foods (91.3 g (95% CI 47.4, 135.1 g) compared with 40 : 60 + rabbit; *p *< 0.001 and 117.7 g (95% CI 73.9, 161.5 g) compared with 10 : 90 + fish; *p *< 0.0001; figure 3*b*).

**Figure 4.**
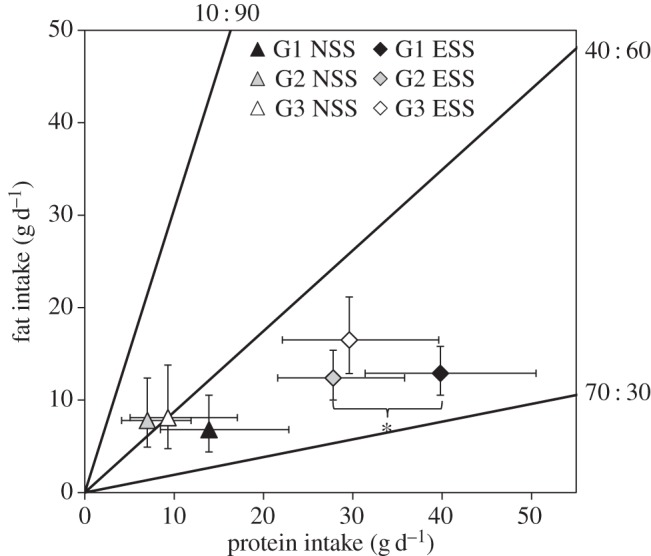
Mean daily protein and fat intake (with 95% CI) for each group of cats during the NSS and ESS of experiment 3. The black lines (nutrient rails) represent the protein : fat balance of each of the foods provided. *Protein intake was significantly different between groups 1 and 2 (*p *= 0.037).

There was no significant effect of group (i.e. foods offered) on either protein (*p *= 0.104) or fat intake (*p *= 0.813) in the NSS phase. Given the difference in total food and energy intake between the NSS and ESS phases, it is no surprise that there was a significant effect of phase on the amounts of protein and fat consumed (*p *< 0.0001 for both macronutrients; figure 4). In the ESS phase, the mixed model analysis revealed there was no significant effect of foods offered (group) on fat intake (*p *= 0.117) or protein intake (*p *= 0.052). Pairwise contrasts between groups showed no significant differences in fat intake (the largest difference was between groups 2 and 3 of 4.1 g, 95% CI −0.6, 7.5 g; *p *= 0.097). There was a significant difference in protein intake between group 1 and 2 (mean difference of 12 g, 95% CI 0.5, 28.1 g; *p *= 0.037), but the difference in protein intake between cats in groups 1 and 3 (mean difference of 10 g, 95% CI −2.1 to 28 g) was not significantly different (*p *= 0.143).

### 3.4. Compilation of experiments 2 and 3

Analysis of the protein and fat intakes averaged over all three groups in the ESS phases of experiments 2 and 3 indicate that cats achieved a consistent balance (ratio) and amounts of protein and fat intake regardless of whether the primary protein source was animal (chicken) or plant (soya) based. Thus, differences in protein and fat intake between the two ESS phases were not statistically significant, amounting to an average of 0.2 g d^−1^ (95% CI −8.6, 12.4 g; *p *= 0.999) for protein and 0.1 g d^−1^ (95% CI −3.3, 4.8; *p *= 0.997) for fat. In experiment 2, protein and fat intake across all three groups averaged 31.8 g d^−1^ (95% CI 25.2, 40.1 g d^−1^) and 13.7 g d^−1^ (95% CI 11.1, 16.8 g d^−1^), respectively, and in experiment 3, it averaged 32.0 g protein d^−1^ (95% CI 25.3, 40.4 g d^−1^) and 13.8 g fat d^−1^ (95% CI 11.2, 17.0 g d^−1^). These protein and fat intakes resulted in an average P : F intake ratio of 1 : 0.430 (F/P = 0.43, 95% CI 0.361, 0.514) and 1 : 0.432 (95% CI 0.361, 0.516) in experiments 2 and 3, respectively, which was not significantly different (average difference of 0.001, 95% CI −0.084, 0.108; *p *= 0.976).

## 4. Discussion

In these studies, we aimed to explore the roles of nutritional and organoleptic properties of food on food choice and macronutrient balancing in a mammalian carnivore, the domestic cat. Previous studies using experimental feeding protocols similar to those used in the present study (i.e. monadic learning and self-selection) have shown that cats balance their macronutrient intake by altering the selection and amounts of foods eaten from the combinations of foods provided [1,20]. Our goal in the present experiments was to challenge the notion that food selection (and the resulting macronutrient intake) may be primarily driven by cats selecting foods that taste nice rather than the result of nutrient balancing. By altering the organoleptic properties of foods with differing macronutrient compositions via the addition of exogenous flavours/aromas (with positive, neutral and negative effects on the palatability of the foods) and standardizing the textural attributes by homogenizing the foods to a ‘porridge-like’ consistency, we could then address the question of whether the organoleptic properties of the foods overrode the mechanisms involved in macronutrient balancing, or vice versa.

The flavours chosen (fish, rabbit and orange) did appear to change the flavour/aroma of the food as perceived by the cats because different amounts of food were consumed depending on the flavour when cats were offered each food individually, with a relative preference of fish > rabbit > orange (experiment 1). The relative preference of the fish flavour was also seen in the NSS phase of experiment 3 where the intake of the fish-flavoured food was greatest regardless of the P : F composition of the foods. However, flavour preferences do not account for the food choices and amounts eaten by cats following the learning period. Rather, our results demonstrate that cats adjusted the choice and amounts of food eaten to achieve a particular nutritional outcome. Using the GF, we see that the balance and amounts of protein and fat consumed converged towards similar points despite the very different P : F compositions and flavour/aroma combinations of the foods offered, indicating regulation towards an intake target for both protein and fat.

This regulation was demonstrated most clearly in the self-selection phase of experiment 2, in which the mean intakes of protein and fat for each group of cats were superimposed on each other (figure 2*b*). In the ESS phase of experiment 3, fat intake did not differ significantly between any of the groups while protein intake was similar between groups 1 and 3 and 2 and 3, but there was a small (approx. 12 g on average), statistically significant difference between groups 1 and 2 (figure 4). This may be simply explained as an influence of the flavour associated with the 70 : 30 foods in these two groups. Thus, for cats in group 1, the association of the preferred fish flavour with the food of highest protein content resulted in a slightly enhanced intake of this food resulting in a marginally greater protein intake particularly compared with cats in group 2 where the food with the same high protein content was associated with the least-preferred flavour (orange). It is not clear why this was evident in experiment 3 compared to experiment 2, but it is possible that the use of soya protein isolate (in experiment 3) rather than chicken breast resulted in a more bland base flavour and the added flavours were therefore relatively stronger in these foods.

The ESS phase of experiment 3 highlights seemingly greater interaction between nutrient content and flavour compared with experiment 2, which impacted on food selection, and that the same nutritional outcome can be achieved through different food choice strategies. Thus, cats in group 3 consumed the 40 : 60 food (which had fish flavour added) in the greatest quantity and there was a compensatory decrease in the amount of the 70 : 30 food consumed relative to the intake of these two foods by cats in group 2 where the amounts of the 40 : 60 and 70 : 30 foods consumed were reversed but with the outcome being that cats in both groups had the same macronutrient intake.

What is remarkable given the unusual nature and properties of the foods offered in these experiments—‘porridge-like’ consistency, added flavours/aromas, different P : F compositions and animal- or plant-derived protein sources—is the extent to which the balance and amounts of protein and fat intakes do converge in experiments 2 and 3 (approx. 32 g protein d^−1^ and 14 g fat d^−1^; P : F intake ratio (grams) of 1 : 0.43). This indicates that macronutrient balancing is a powerful driver of food selection in cats and points to the ability to detect and respond to post-ingestive macronutrient signals that are distinct from sensory aspects contributing to the apparent palatability of foods. The protein and fat intakes in the present study are also very similar to those seen in previous studies in cats in which the foods offered were typical of commercially available wet cat food products (see fig. 8 in [1]) or wet and dry products (see table 2 in [20]) and indicate that the ability to regulate macronutrient intake in cats is evident even when the foods provided differ in various properties including moisture content, texture, macronutrient composition and flavour/aroma.

Based on Atwater factors of 16.7 and 37.6 kJ g^−1^ for protein and fat, respectively, the average amounts of protein and fat consumed by the cats in the present studies would provide approximately equal energy from each macronutrient. Given that the foods contained negligible energy from carbohydrate, this would equate to approximately 50% of total daily energy intake from protein and 50% from fat which is very similar to the 52 : 46 : 2 (per cent energy from protein : fat : carbohydrate, respectively) dietary composition estimated for feral cats [33]. The amounts of protein and fat ingested by cats appear similar to those reported for farmed mink (*N. vison*; [2]). As obligate carnivores that have evolved on a prey-based diet consisting primarily of protein and fat, it can be hypothesized that both these species have similar metabolic pathways and requirements that are best met through regulating their intake of these two macronutrients to similar ratios and amounts. It would be interesting to know if a similar balance of P : F intake is common to other wild vertebrate hypercarnivores (mink, polar bears, tigers, etc.) and warrants further investigation.

The introduction of the naive selection phase in experiment 3 indicated that the initial intake of the foods was driven primarily by organoleptic properties (i.e. flavour/aroma) given that intake of the fish-flavoured food within each group of cats was greatest regardless of the PE : FE composition of the food. It also highlighted that food and energy intake was approximately half that seen in the ESS phase. This probably reflects that the ‘porridge-like’ format of the food was very different from the standard commercial foods usually provided to the cats and that it took some time for the cats to become familiar with and accepting of the foods. Exposing the cats to the learning phase during which each PE : FE–flavour combination for their group was presented alone appeared to facilitate the ability of cats to distinguish the nutritional aspects of the foods from the organoleptic properties. When presented with the choice of foods following the learning phase, the patterns of food intake were certainly not aligned with the order of preference for the added flavours, particularly in the case of cats in group 2 where the food (i.e. 70 : 30) combined with the least-preferred flavour (orange) was consumed in the greatest quantity. In addition, the use of soya protein isolate as the major protein source in the foods offered in experiment 3 indicated that the significantly greater intake of the 70 : 30 food in each of the groups in experiment 2 was not the result of some potential organoleptic signal from the high inclusion of chicken breast in this food, because the intake of the 70 : 30 food was also greatest for cats in group 1 and 2 in experiment 3.

The results of our study clearly demonstrate that organoleptic properties (e.g. flavour or aroma) and macronutrient composition play nominally independent roles in diet selection by cats, and the data also suggested that in some cases they might interact in their effects. The most important findings, however, are that even though organoleptic properties might override in the short term, with experience, macronutrient regulation prevails. This reinforces the important role that macronutrition can play in diet selection by obligate predators, a proposition that until recently has been doubted by many [18,35]. One grand challenge for future research is to determine the physiological mechanisms which underpin the apparent regulation of macronutrient intake which currently are poorly understood [21,36]. From the broader perspective of carnivore nutritional ecology, our results raise the important question of whether and how these factors influence prey selection by predators in the wild.

## Supplementary Material

Self-selection data: This spreadsheet contains the food and macronutrient intake data from cats during self-selection phases of experiments 2 and 3.
